# The Influence of Biological Sex on Presentation and Outcomes of Acute Myocarditis: A Systematic Review and Meta-Analysis

**DOI:** 10.7759/cureus.57325

**Published:** 2024-03-31

**Authors:** Meghana C Muppuri, Lavanya Gopinath, Zainab Tariq, Sabina Shah, Rafael Cortorreal Javier, Fizza Mahmood, Dhruvi Modi, Maria Joseph, Ravikishore Reddy Gopavaram, Shriya Sharma, Mohammed Al-Tawil

**Affiliations:** 1 Internal Medicine/Radiology, Bioprist Institute of Medical Sciences, Montego Bay, JAM; 2 Radiology, University of Alabama at Birmingham, Birmingham, USA; 3 Internal Medicine, Bangalore Medical College and Research Institute, Bangalore, IND; 4 Cardiology, Khyber Medical College, Peshawar, PAK; 5 Internal Medicine, Nepal Medical College and Teaching Hospitals, Kathmandu, NPL; 6 Medicine, Universidad Iberoamericana (UNIBE), Santo Domingo, DOM; 7 Cardiology/Cardiac Surgery, Shifa College of Medicine, Shifa Tameer-e-Millat University, Islamabad, PAK; 8 Internal Medicine, Gujarat Adani Institute of Medical Sciences, Bhuj, IND; 9 Internal Medicine, Odessa National Medical University, Odessa, UKR; 10 Internal Medicine, Dnipro State Medical University, Dnipro, UKR; 11 Cardiology, Al-Quds Hospital, Jerusalem, PSE

**Keywords:** meta-analysis, gender, acute myocarditis, myocarditis, sex-related differences, pathophysiology, outcomes, systematic review

## Abstract

There is growing evidence of sex-related differences in the epidemiology and pathophysiology of cardiovascular diseases. This is the first systematic review and meta-analysis that aimed to highlight the sex-specific differences in the clinical features and outcomes of acute myocarditis. Electronic searches were performed on Scopus, Embase, and PubMed from inception up to June 2023 to identify studies comparing the clinical features and outcomes of acute myocarditis in males and females. Both qualitative and quantitative summaries were conducted.

In this systematic review and meta-analysis of 11 studies involving 34,791 patients presenting with acute myocarditis. Male patients, who comprised 69.8% of the entire pooled population, presented at a markedly younger age (mean difference: -8.99 years; 95% CI: -13.60, -4.38; p=0.0001). They also had significantly lower rates of hypertension, diabetes mellitus, and coronary artery disease compared to female patients (p<0.01). Male patients were more likely to present with ST elevation (RR: 2.57 [1.38, 4.79]; p=0.003) and higher troponin levels (standardized MD: 0.79; 95% CI: 0.43, 1.15; p<0.0001) compared to female patients. This review underscores the crucial sex-specific evaluation in acute myocarditis, necessitating tailored approaches in assessment and diagnostic evaluation, and emphasizing the need for additional research in this domain.

## Introduction and background

Myocarditis refers to the inflammation of the myocardium, which is the muscular middle layer of the heart wall responsible for its contraction. This inflammation typically results from viral infections such as coxsackievirus and parvovirus, autoimmune disorders, or exposure to certain toxins [1,2]. Unlike several other cardiovascular diseases, old age is not a risk factor for myocarditis [1]. Moreover, the Global Burden of Disease 2016 and 2019 Studies (GBD2016 and GBD2019) show a marked increase in all-age deaths due to myocarditis over the last decades [2]. There is growing evidence of sex-related differences in the epidemiology and pathophysiology of cardiovascular diseases, with females experiencing a more favorable natural history for several cardiovascular conditions than males. Furthermore, a recent finding reports a female-to-male ratio between 1:1.5 and 1:1.7 in patients with myocarditis [3]. Current studies have also highlighted numerous sex differences in clinical characteristics, management, resource utilization, and long-term outcomes of myocarditis [1-4]. For instance, females remain at high risk for myocarditis-related complications and in-hospital mortality [4], since females usually exhibit stronger innate and adaptive immune responses than males [3].

A better understanding of existing literature on the impact of sex on disease could aid in the sex-specific adaptation of diagnosis and treatment. To our knowledge, no previous meta-analysis has assessed the association between biological sex and myocarditis. Accurately identifying sex-specific risk and protective factors for myocarditis is vital to its treatment. Therefore, we undertook a meta-analysis of the literature aiming to assess the association between biological sex and myocarditis and analyze the impact of biological sex on clinical characteristics, management, and outcomes of myocarditis.

## Review

Materials and methods

Literature Search

Electronic searches were performed on Scopus, Embase, and PubMed from inception to June 2023. Search terms were formulated using the Population, Intervention, Comparison, and Outcome (PICO) framework to identify studies comparing acute myocarditis in males and females. This study adhered to the updated 2020 version of the Preferred Reporting Items for Systematic Reviews and Meta-Analyses (PRISMA) guidelines [5]. The keywords employed are as follows: (Myocarditis [title]) AND (sex OR gender [title/abstract]). To ensure a comprehensive search and inclusion of all the relevant literature, meticulous forward and backward citations were conducted. The results were initially screened through the titles and abstracts by independent authors. Any conflicts were resolved through review by another author. The final selection of the studies used the pre-established inclusion and exclusion criteria.

Studies Selection

To be included, studies had to be observational, retrospective cohort studies (in English) with at least 10 human myocarditis patients that compared differences based on sex or gender stratification and reported either one or all the following treatment outcomes: presenting symptoms, ECG changes, and cardiac biomarkers. Studies with full-text availability were included. Systematic reviews, meta-analyses, narrative reviews, case reports/series, editorials, study protocols, abstracts, commentaries, letters to the editor, and the studies that report outcomes for only one sex or fail to compare outcomes with other sex, and studies specifying patients with age <18 years were excluded from this review.

Data Extraction and Quality Assessment

Two independent investigators were responsible for data extraction from each included article. Two other members further revised the obtained data, and any conflicts were resolved to ensure consistency and accuracy. The extracted data focused on study characteristics and key demographic data, including age, sex, and the occurrence of any comorbidities, such as diabetes mellitus (DM), hypertension (HTN), cerebral artery disease (CAD), stroke, and autoimmune disease. Extraction also focused on fever and ECG changes, such as atrial fibrillation, bradycardia, tachycardia, and troponin levels. Additionally, in-hospital stay after treatment and interventions, such as prior implantable cardioverter-defibrillators (ICD), were assessed. Categorical data were extracted as events and the total for each group, while continuous data were coded as mean and standard deviation. If the data were reported in other formats, the method by Wan et al. was used to perform necessary conversions [6]. The main outcomes included incidence, age of the patient, biomarkers such as troponin and C-reactive protein levels, ECG changes, and short-term and long-term complications resulting from acute myocarditis. The secondary outcomes included cardiac function, such as differences in ejection fraction, ventricular dilation, and contractility. A quality appraisal schema based on the Newcastle-Ottawa Scale (NOS) risk of bias assessment tool was used to assess all included studies. Two reviewers independently assessed the risk of bias, and a final table was assembled based on their agreement. The evaluation cut-off for the follow-up length was set at 30 days. Follow-up was considered sufficient if no more than 10% of the patient cohort data were lost.

Statistical Analysis

This meta-analysis was conducted in accordance with the guidelines outlined by the Cochrane Collaboration and the Meta-analysis of Observational Studies in Epidemiology (MOOSE) [7]. Data analysis was performed using Review Manager Software version 5.4.1 (London, England: Cochrane Foundation). The Mantel Haenszel random effects model was applied to calculate the risk ratio (RR) with the corresponding 95% confidence intervals (CI) for binary outcome measures. To evaluate the presence of statistical heterogeneity, we utilized Cochrane's Q-test for heterogeneity and I^2^ statistics. An I^2^ value exceeding 50% was considered indicative of substantial heterogeneity among the included studies. Statistical significance was defined by a p-value below 0.05. In order to ascertain the robustness of the findings, a sensitivity analysis was conducted. This analysis involved examining the impact of individual studies on the overall results.

Results

Characteristics of the Included Studies

**Figure 1:**
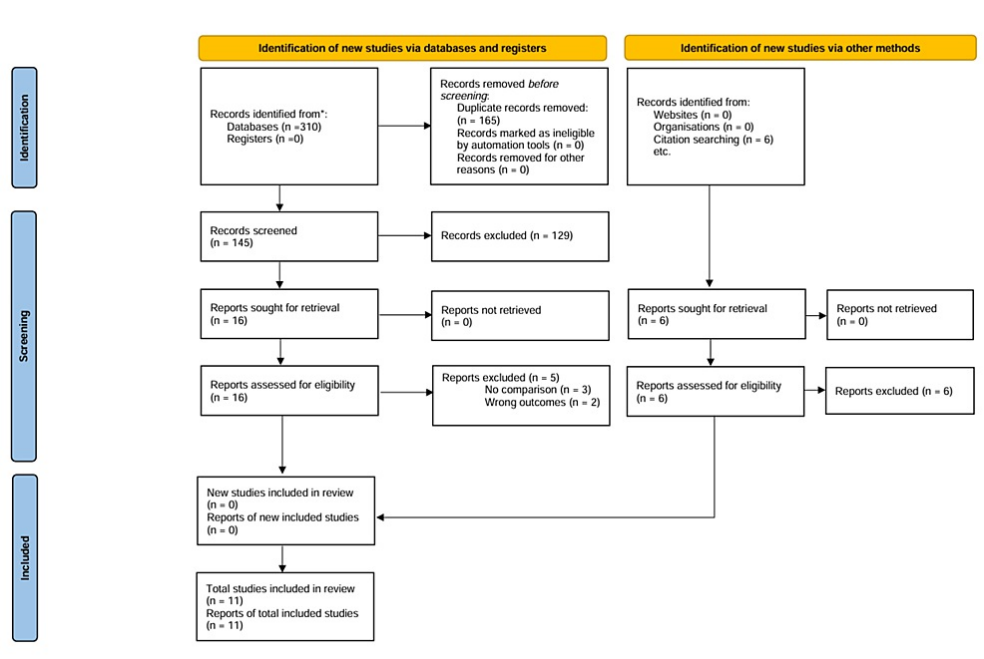
The PRISMA flowchart illustrating the different phases of screening. PRISMA: Preferred Reporting Items for Systematic Reviews and Meta-Analyses

Ultimately, we included a total of 11 studies [1-3,8-15] in our systematic review and nine studies in the meta-analysis. The PRISMA flowchart is presented in Figure 1. Eleven studies involved 34,791 patients presenting with acute myocarditis. Male patients comprised 69.8% of the entire pooled population.

The studies provided sex-stratified patient outcomes following acute myocarditis. Characteristics of the included studies as well as their main findings are summarized in Table 1. All the studies were assessed using the NOS tool and results were displayed in Table 2. The quality of the included studies ranged between moderate and good quality.

**Table 1:** Summary of study characteristics. CRP: C-reactive protein; CMR: cardiac magnetic resonance; LGE: late gadolinium enhancement

S. no.	Study ID	Location	Study duration	Study design	Number of patients		Study main finding
					Male	Female	
1	Mirna et al. 2022 [1]	Austria	2009-2019	Retrospective	174	50	Female patients were older, had a higher prevalence of respiratory tract infections, and less frequent presentation with ST elevation. CRP was lower in females and they were less likely to undergo CMR.
2	Ozieranski et. al 2022 [2]	Poland	2011-2019	Retrospective	12,111	4,208	The incidence of acute myocarditis increased over time in males. Females presented with more symptoms and had more comorbidities. Females presenting at ages 20-40 years had a significantly poorer prognosis than males of the same age.
3	Castrichini et al. 2022 [3]	Italy	2005-2019	Retrospective	211	101	Females experienced a more favorable long-term prognosis than males, despite a similar clinical profile at presentation.
4	Kytö et al. 2013 [8]	Finland	2000-2009	Retrospective	2,450	748	Males (younger) are more prone to myocarditis than females. Females are most affected post-menopause. Hospital admissions for myocarditis decrease with age.
5	Cherian et al. 2018 [9]	USA	2008-2010	Retrospective	644	346	Females were older with more comorbidities upon admission. They had a notably higher in-hospital mortality than males.
6	Wong and Christiansen 2017 [10]	New Zealand	2007-2016	Retrospective	126	52	Males showed a clear inclination, with differences in symptoms and biomarker levels. Imaging use was consistent regardless of ST elevation presence.
7	Patriki et al. 2020 [11]	Switzerland	2011-2018	Retrospective	41	10	Like myocardial infarction, atypical symptoms might lead to underdiagnosis and underrepresentation of females in myocarditis studies.
8	Elzanaty et al. 2024 [12]	USA	2016-2019	Retrospective	8,113	4,884	Females admitted with myocarditis have similar in-hospital outcomes with higher risk of readmission within 90 days from hospitalization.
9	Younis et al. 2020 [13]	Israel	2005-2017	Retrospective	272	50	Male patients were younger, more likely to present with ST elevation, had higher troponin levels at admission, and higher rate of ventricular arrhythmias compared to females. There were no differences in post-discharge mortality rates between males and females.
10	Cocker et al. 2009 [14]	Canada	2006-2009	Prospective	41	24	Myocardial fibrosis was common in males and those under 40 years of age. Younger patients experienced more regional injury, due to a higher rate of irreversible damage.
11	Cau et al. 2023 [15]	Italy	2017-2023	Retrospective	102	33	Females show more septal LGE involvement, regardless of age, cardiovascular risks, or CMR metrics.

**Table 2:** Risk of bias assessment using the Newcastle-Ottawa Scale. Follow-up length was determined to be 12 months and adequacy of follow-up meant less than 10% loss at 12 months. Stars (★) denote that the study fulfills the criteria for this assessment item, whereas a zero signifies the absence of that measure in the study's design.

Study ID	Selection				Comparability		Outcome			Score
	Representativeness of the exposed	Selection of non-exposed	Ascertainment of exposure	Outcome of interest not present at start	Main factor	Additional factor	Assessment	Follow-up length	Adequacy of follow-up	
Mirna et. al [1]	★	★	★	★	★	★	★	★	★	9
Ozieranski et. al [2]	★	★	★	★	★	★	★	★	★	9
Castrichini et. al [3]	★	★	★	★	★	★	★	★	★	9
Kytö et. al [8]	★	★	★	★	★	★	★	0	0	7
Cherian et. al [9]	★	★	★	★	★	0	★	0	0	6
Wong and Christiansen [10]	★	★	★	★	★	0	★	0	0	6
Patriki et. al [11]	★	★	★	0	★	★	★	0	0	6
Elzanaty et. al [12]	★	★	★	★	★	★	★	0	0	7
Younis et. al [13]	★	★	★	★	★	★	★	★	★	9
Cocker et. al [14]	★	★	★	★	★	0	★	0	0	6
Cau et. al [15]	★	★	★	★	★	★	★	0	0	7

Demographic Characteristics

**Figure 2:**
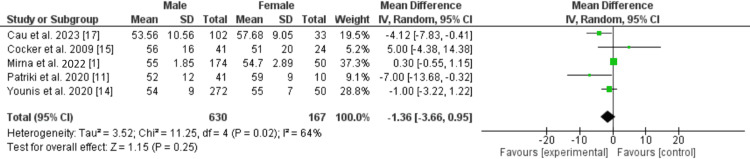
Difference in age of patients presenting with acute myocarditis.

Analysis of demographic characteristics showed that male patients presented at a markedly younger age (mean difference: -8.99 years; 95% CI: -13.60, -4.38; p=0.0001), were less likely to have heart failure (RR: 0.60; 95% CI: 0.41, 0.86; p=0.005), hypertension (RR: 0.62; 95% CI: 0.43, 0.89; p=0.009), and diabetes (RR: 0.54; 95% CI: 0.33, 0.87; p=0.01) and a history of coronary artery disease (RR: 0.35; 0.13, 0.98; p=0.05) compared to females with acute myocarditis (Figure 2) [1-3,8,11-15]. Additionally, male patients had a higher left ventricular end-diastolic diameter (LVEDD) than female patients (MD: 5.16 mm; 95% CI: 3.29, 7.03; p<0.00001) at the diagnosis of acute myocarditis. Table 3 and Table 4 summarize the pooled results of the meta-analysis.

**Table 3:** Meta-analysis of sex-specific co-morbidity profile in patients presenting with acute myocarditis. *Only reported by two studies with small samples. **Statistically significant value. ***Borderline significance value.

Sex-specific comorbidities	Males	Females	Risk ratio (95% CI)	p-Value
Heart failure	5%	10%	0.60 (0.41, 0.86)	0.005**
Hypertension	16%	35%	0.62 (0.43, 0.89)	0.009**
Diabetes	4%	8%	0.54 (0.33, 0.87)	0.01**
History of coronary artery disease	5%	12%	0.35 (0.13, 0.98)	0.05***
Previous stroke	1%	3%	0.29 (0.05, 1.64)	0.16
Atrial fibrillation*	13%	26%	0.62 (0.19, 2.03)	0.43

**Table 4:** Meta-analysis of continuous outcomes in patients presenting with acute myocarditis. *P-value <0.05 is considered statistically significant. Effect estimate is presented as mean difference (MD) calculated using inverse variance (IV), random effects model for continuous variables. LVEF: left ventricular ejection fraction; LVEDD: left ventricular end-diastolic diameter

Outcomes (mean difference)	Effect measure	95% CI	p-Value
Age (ref. female)	-8.99 years	-13.60, -4.38	0.0001*
LVEDD	5.16 mm	3.29, 7.03	<0.00001*
LVEF%	- 1.36%	-3.65, 0.92	0.24
Laboratory results (Std. mean difference)			
C-reactive protein	0.77	-0.98, 2.51	0.39
Troponin levels	0.79	0.43, 1.15	<0.00001*

**Figure 3:**
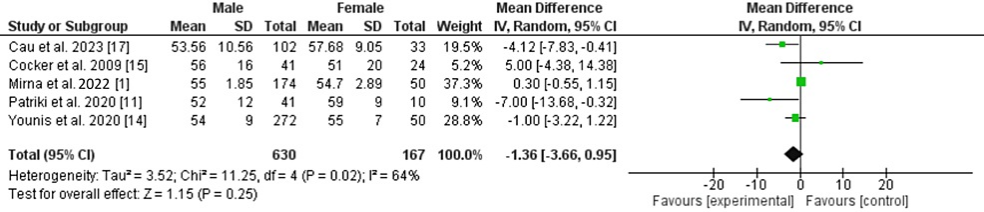
LVEF at presentation in patients with acute myocarditis. LVEF: left ventricular ejection fraction

There was no significant difference in risk of stroke (RR: 0.29 [0.05, 1.64]; p=0.16), or atrial fibrillation (RR: 0.62 [0.19, 2.03]; p=0.43) between male and female patients with acute myocarditis. There was also no significant difference in left ventricular ejection fraction % (LVEF%) at presentation between male and female patients with myocarditis (MD: -1.36 [-3.65, 0.92]; p=0.24; I=64%) as shown in Figure 3 [1-3,8,11-15].

Clinical Features and Diagnostics

**Figure 4:**
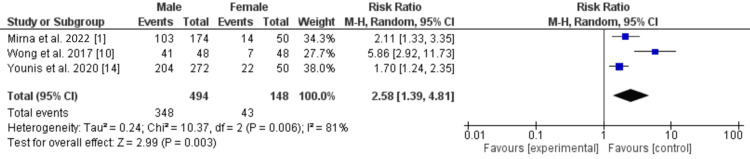
ST elevation at presentation in patients with acute myocarditis.

**Figure 5:**
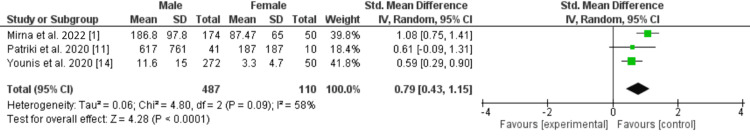
Troponin levels at presentation in patients with acute myocarditis.

Male patients were more likely to present with ST elevation (RR: 2.57; 95% CI: 1.38, 4.79; p=0.003) (Figure 4) [1,10,13], and significantly higher troponin levels (standardized MD: 0.79; 95% CI: 0.43, 1.15; p<0.0001) (Figure 5) [1,11,13]. Male patients also had a higher C-reactive protein level (standardized MD: 0.77 [-0.98, 2.51]; p=0.39; I^2^=0%) at presentation. However, the results were not statistically significant.

There was no significant difference in late gadolinium enhancement rates in cardiac magnetic resonance (CMR) (RR: 1.09; 95% CI: 0.45, 2.64; p=0.84; I^2^=90%), the indication for endomyocardial biopsy (RR: 1.10, 95% CI: 0.11, 11.33; p=0.93; I^2^=91%) and rates of coronary angiography (RR: 2.25, 95% CI: 0.55, 9.25; p=0.26, I^2^=92%) between male and female patients with acute myocarditis.

Short-term all-cause mortality risk was similar between males and females (RR: 1.11 [0.27, 4.56]; p=0.89) following acute myocarditis. There was no significant difference in all-cause mortality rates during long-term follow-up (>30 days) between the two groups (RR: 1.26 [0.20, 7.84] p=0.80).

Discussion

Given its considerable etiologies, the presentation and disease severity of acute myocarditis show substantial variation. In our detailed analysis of 11 studies consisting of the 34,791 patients included in this systematic review and meta-analysis, the prevalence of acute myocarditis is notably more significant in males than in females (69.9% vs. 30.1%). The earlier onset of acute myocarditis in males compared to females likely arises from the interplay of elevated testosterone levels in males, while the delayed presentation in females may be attributed to declining estrogen levels typically occurring around menopause [8]. Moreover, males were reported to be less likely to have comorbidities, which supports the influential role of testosterone in the development of this condition. In addition to this, the presence of comorbidities may influence long-term outcomes, more particularly in females as they present with older age, and more commonly postmenopausal [8]. Female patients tended to present with atypical course in comparison to males, and with an associated delay in diagnosis and treatment. These include the presence of multiple co-morbidities and the increased difficulty in obtaining the right diagnosis. This emphasizes the significance of variation in disease severity and presentation. To the best of our knowledge, the present analysis represents the first attempt to comprehensively assess the impact of sex on pathophysiology, clinical management and treatment, the outcomes, as well as origins of myocarditis [2,11,12,16].

This sex-based disparity in incidence extends to the age of onset, with males presenting at a younger age. This age difference may be attributed, in part, to the influences of testosterone elevation and estrogen decline, as these hormonal changes can potentially impact the development of myocarditis [8,11,12]. Testosterone increases viral binding to myocytes, eliciting a Th1-type immune response and inhibition of anti-inflammatory cells [8]. Estrogen, on the other hand, has been shown to have inhibitory effects by favoring the inhibition of pro-inflammatory T cells [13]. Given that the majority of the female patients presented with acute myocarditis at an older age, declining levels of estrogen and higher levels of testosterone may have contributed to the process. Additionally, males were less likely to have comorbidities, further suggesting the influential role of testosterone in the pathogenesis of myocarditis. Conversely, female patients tended to present with an atypical disease course including delayed presentation and more frequent comorbidities compared to males, often leading to delayed diagnosis and treatment. These findings underscore the substantial variations in disease severity and presentation, emphasizing the importance of recognizing these sex-specific differences.

Mirna et al. [1] concluded that female patients were more likely to be underdiagnosed or have delayed work-up. However, we did not find statistically significant differences in the indications for CMR, endomyocardial biopsy, or coronary angiography in our analysis. Importantly, these analyses had severe heterogeneity. The heterogeneity in cardiac magnetic resonance, endomyocardial biopsy, and coronary angiography results can be attributed to between-center differences in protocols of management and the wide span of the study periods [1,17].

Our analysis has yielded the conclusion that the short-term risk of all-cause mortality (within 30 days) is comparable between both groups. Cocker et al.'s study has suggested that young age serves as a predictor of death and transplantation in patients with biopsy-confirmed myocarditis [14]. Moreover, fatal myocarditis appears to be most prevalent among individuals under the age of 40 years. In contrast, males are known to have a higher incidence of mortality from myocarditis [14]. The study conducted by Ozierański et al. has indicated that female patients in the age range of 20-40 years exhibit a significantly poorer prognosis compared to their male counterparts within the same age group [2]. Such results concord with the findings of more severe disease and higher mortality rates in younger populations [15]. Perhaps, the combination of a young age in males and comorbidities in females balances out the observed risk of mortality in registries’ data included in our analysis.

The incidence of ST elevation and troponin levels was notably higher in males. Additionally, males presented with larger left ventricular end-diastolic diameters (LVEDDs). However, it's worth noting that the higher LVEDD in males may be attributed to their greater body surface area rather than being directly associated with the presence of myocarditis [1].

In terms of electrocardiograms (ECGs), our observations revealed a lower occurrence of ST-segment elevations in females compared to males. This finding aligns with research by Younis et al., which indicated that males are less likely to present with a normal ECG compared to females [13].

Cardiovascular magnetic resonance (CMR) utilizing late gadolinium enhancement (LGE) is a non-invasive imaging technique employed to assess myocardial tissue composition, particularly myocardial fibrosis [15]. LGE is a robust and independent risk factor for ventricular arrhythmias in dilated cardiomyopathy [13]. Furthermore, during the acute phase of acute myocarditis, LGE can indicate myocardial fibrosis, necrosis, and inflammation [15]. Its presence in cardiac magnetic resonance imaging has also been identified as a strong predictor of mortality and cardiovascular risk [2].

Despite the absence of significant differences in the extent of LGE in our analysis, a study by Cau et al. discovered that females were more likely to exhibit greater involvement of LGE in the septal myocardium, despite a higher prevalence of myocarditis in males. Moreover, the cardiovascular risk factor profiles between the sexes were similar [15]. Additionally, Ozierański et al. demonstrated that mid-wall LGE in the (antero-) septal segments was linked to a higher incidence of death, including sudden cardiac death, during a follow-up period exceeding 10 years in patients with biopsy-proven myocarditis [2]. Therefore, it is reasonable to speculate on how the association between sex and the presence, extent, and location of LGE might influence the pathophysiology of myocarditis in future research. Indeed, considering the extent of LGE may be a pivotal variable in future investigations.

Based on the findings from our study, it is beneficial to underscore the need for further research into this topic. The exact roles of testosterone and estrogen in acute myocarditis along with the interplay between myocarditis and comorbidities demand a deeper exploration as they hold significant value in outcomes and prognosis. The different etiologies of myocarditis and how they manifest across sexes, along with the disparity in age at presentation can offer invaluable information once further studied. More insights into these aspects can revolutionize the approach to acute myocarditis and specific goal-directed diagnosis and treatment.

Strengths and limitations

Our study's strengths lie in its comprehensive assessment, large sample size, inclusiveness, implications for future research, and addressing a notable gap in the existing literature. By comprehensively examining various facets of acute myocarditis, including prevalence, clinical presentation, comorbidities, diagnostics, and short-term mortality, we provide a holistic understanding of this complex condition and the need for future research. Our findings set the stage for future investigations into sex-specific tailored diagnostic and treatment approaches.

However, our study is not without limitations. Heterogeneity in study populations, potential publication bias, variable data quality, different hospital protocols, temporal changes in diagnostic and treatment approaches over time, and the possibility of uncontrolled confounders may introduce bias and variability into our results. Also, we did not incorporate unpublished studies, which also limited the generalizability of our findings. Despite these limitations, our study represents a critical step in shedding light on sex-specific nuances in acute myocarditis and emphasizes the need for further research to improve patient care. The observational and retrospective nature of the included studies necessitates caution when trying to generalize the results of this meta-analysis.

Future directions

Based on our study results, there is a need for further exploration into the influence of sex on the physiological and clinical course as well as the prognosis of acute myocarditis. Additionally, it is imperative to develop awareness programs tailored to sex-specific management approaches for healthcare providers and to conduct well-planned clinical trials that take into account sex-specific analysis. These efforts are essential for advancing the field, improving treatment outcomes, and promoting equal care for all patients.

## Conclusions

Our study reveals significant sex-based disparities in the prevalence, presentation, and clinical outcomes of acute myocarditis. Males exhibit a higher prevalence and younger age of onset, possibly linked to testosterone's influence. Females often present with atypical symptoms, leading to diagnostic delays. Despite sex-specific differences, short-term mortality rates are comparable. These findings underscore the importance of considering sex in myocarditis research and clinical management, highlighting the need for tailored diagnostic and treatment approaches.
