# Correction: The Influence of Biological Sex on Presentation and Outcomes of Acute Myocarditis: A Systematic Review and Meta-Analysis

**DOI:** 10.7759/cureus.c180

**Published:** 2024-06-06

**Authors:** Meghana C Muppuri, Lavanya Gopinath, Zainab Tariq, Sabina Shah, Rafael Cortorreal Javier, Fizza Mahmood, Dhruvi Modi, Maria Joseph, Ravikishore Reddy Gopavaram, Shriya Sharma, Mohammed Al-Tawil

**Affiliations:** 1 Internal Medicine/Radiology, Bioprist Institute of Medical Sciences, Montego Bay, JAM; 2 Radiology, University of Alabama at Birmingham, Birmingham, USA; 3 Internal Medicine, Bangalore Medical College and Research Institute, Bangalore, IND; 4 Cardiology, Khyber Medical College, Peshawar, PAK; 5 Internal Medicine, Nepal Medical College and Teaching Hospitals, Kathmandu, NPL; 6 Medicine, Universidad Iberoamericana (UNIBE), Santo Domingo, DOM; 7 Cardiology/Cardiac Surgery, Shifa College of Medicine, Shifa Tameer-e-Millat University, Islamabad, PAK; 8 Internal Medicine, Gujarat Adani Institute of Medical Sciences, Bhuj, IND; 9 Internal Medicine, Odessa National Medical University, Odessa, UKR; 10 Internal Medicine, Dnipro State Medical University, Dnipro, UKR; 11 Cardiology, Al-Quds Hospital, Jerusalem, PSE

 

This article has been corrected as follows:

The earlier version of the abstract mentioned that males had higher C-reactive protein levels (RR: 3.04 [2.75, 3.34]; p<0.00001) compared to female patients. This was incorrect and has been corrected to: higher troponin levels (standardized MD: 0.79; 95% CI: 0.43, 1.15; p<0.0001).

The authors and journal deeply regret that this error was not identified and addressed prior to publication.

 

